# Endocarditis and other indications for open-heart surgery after a transcatheter aortic valve implant

**DOI:** 10.1093/icvts/ivaf173

**Published:** 2025-07-26

**Authors:** Torbjörn Ivert, Aninda Omar, Andreas Rück, Magnus Dalén

**Affiliations:** Department of Cardiothoracic Surgery, Karolinska University Hospital and Molecular Medicine and Surgery, Karolinska Institutet, 17177 Stockholm, Sweden; Division of Cardiology, Department of Medicine, Karolinska Institutet, 17177 Stockholm, Sweden; Division of Cardiology, Department of Medicine, Karolinska Institutet, 17177 Stockholm, Sweden; Department of Cardiothoracic Surgery, Karolinska University Hospital and Molecular Medicine and Surgery, Karolinska Institutet, 17177 Stockholm, Sweden

**Keywords:** TAVI, endocarditis, valve dysfunction

## Abstract

This retrospective observational single-centre analysis included 31 patients who underwent transcatheter aortic valve implantation (TAVI) between 2016 and 2024 and subsequent open-heart surgery between 2019 and 2024. They were admitted from infectious disease departments or cardiology clinics and accounted for 0.7% of heart operations performed in 2024. The incidence of definite endocarditis was 0.5% (17/3226) of all TAVI procedures performed during this period. Of the 17 patients, 9 (53%) with definite endocarditis underwent aortic valve replacement, with 1 early death (11%) from bowel ischaemia and liver failure. The 2-year postoperative survival for definite endocarditis was 76%. Open-heart surgery was contraindicated in all 8 patients with definite endocarditis due to severe comorbidities and frailty. These patients died within 2 years of the infection or due to heart failure. Furthermore, 10 patients classified as having endocarditis after TAVI were medically treated and had a 2-year survival rate of 72%. The survival rate was 87% at 2 years after open-heart surgery for non-infectious indications performed up to 5 years after TAVI in 22 patients. In conclusion, heart surgery can be curative in selected patients with definite endocarditis after TAVI and lifesaving after rare TAVI complications.

## INTRODUCTION

The first transcatheter aortic valve implant (TAVI) in Sweden was performed in 2008 at the Karolinska University Hospital in Stockholm. A TAVI was initially reserved for elderly patients with aortic stenosis who were not candidates for open aortic valve replacement because of fragility and comorbidities. Cardiac surgery is also not an option in cases of procedural complications, such as late valve dysfunction or infection. Recently, the PARTNER 3 and Evolut Low-Risk trials have shown that the outcomes after TAVI are not inferior to those after surgical aortic valve replacement (SAVR) in patients with low or intermediate risk.^1^^,^^2^ Low mortality, a reduced need for permanent pacemakers, and fewer paravalvular leaks have widened the indications for TAVI among younger patients, especially those with a history of cardiac operations and those who prefer TAVI to SAVR. According to the Society of Thoracic Surgeons Adult Cardiac Surgery Database, the incidence of cardiac surgery after TAVI is increasing.^3^ The most rapidly growing operation involves prosthesis explants. Additionally, SAVR after TAVI has been reported to have high operative mortality. To date, few reports have described open-heart surgery performed after TAVI for endocarditis.^4–6^ This study aimed to evaluate the incidence, indications, and outcomes of open-heart surgery after TAVI.

## PATIENTS AND METHODS

This study is a retrospective, observational, single-centre analysis of patients who underwent open-heart surgery after a previous TAVI. The decision to perform TAVI was made in a multidisciplinary team meeting. All 31 patients underwent TAVI between 2016 and 2024 and open-heart surgery between 2019 and 2024. Intravenous antibiotic prophylaxis (2 g isoxazolyl penicillin 30 min before TAVI and repeated after 4 and 8 h) was administered. The patients were admitted to infectious disease departments or cardiology clinics. All patients were provided written information and gave verbal consent that data from the operation nay be reported to national registers and may be used for research. Data were anonymized before analysis. The Swedish Ethical Review Authority (Dnr. 2024-00542-01) approved this study.

Cardiac surgery risk was assessed using the European System for Cardiac Operative Risk Evaluation Score (EuroSCORE) II. Follow-up ended on 31 January 2025.

### Statistical methods

Continuous data are presented as medians with ranges. Analysis of variance was used to compare 3 groups. The Kaplan–Meier method was used to calculate cumulative survival.

### Definitions

Endocarditis was classified as possible or definite according to the modified Duke criteria.^7^ The first admission date for assumed TAVI endocarditis was used to calculate the time from the onset of infection. All patients with endocarditis were evaluated using transoesophageal echocardiography. Definite endocarditis was defined as a positive blood culture, valve vegetation, or findings of infection confirmed during surgery. Patients with a previous TAVI who had a febrile course and septicaemia with a positive blood culture, but no cusp vegetation, were classified as having possible endocarditis, and treated with intravenous antibiotics for several weeks.

## RESULTS

The first open-heart operation after a previous TAVI was performed in 2019. By 2024, the number increased to 8. This accounted for 0.7% of the 1070 heart operations (**Figure S1**). Patient details are listed in **Tables S1 and S2**.

### Endocarditis

We identified 17 and 10 patients with definite and possible endocarditis, respectively. The patient characteristics and blood culture results are listed in **Table 1**. Of all the patients who underwent TAVI between 2016 and 2024, the incidence of definite endocarditis was 0.5% (17/3226).

**Table 1.** Indications for open-heart surgery in patients with a previous transcatheter aortic valve implant

Indication for surgery	Number of operations	Definite endocarditis		Possible endocarditis	*P-* value
		Surgery performed (*n *= 9)	Medical treatment (*n *= 8)	Medical treatment (*n *= 10)	
Endocarditis	9	Median (range)	Median (range)	Median (range)	
Age at TAVI (years)		74 (61-81)	78 (5-88)	81 (66-88)	0.14
Time from TAVI until endocarditis (months)		16 (1-83)	10(1-42)	9 (1-187)	0.69
EuroSCORE II, mean (95% CI)		*10.4 (1.1-73.7)*	*8.1 (3.2-15.4)*	*6.1 (3.9-11.0)*	0.78
		*n* (%)	*n* (%)	*n* (%)	
Females		1 (11)	4 (50)	2 (20)	
Onset of infection < 12 months		3 (33)	3 (38)	6 (60)	
No. of previous interventions		1 PCI, 1 CABG	1 SAVR, 1 PCI	1 SAVR	
Blood cultures					
*Streptococcus mitis*		*6*	*3*	*5*	
*Staphyloccous aureus*			*4*	*2*	
*Enterococcus faecalis*		*4*		*3*	
*Staphylococcus epidermidis*			*1*		
Non-infectious					
Structural valve deterioration	3	1 cusp rupture, 2 aortic stenosis			
Non-structural valve cause	10	5 malpositioning of prosthesis, 2 paravalvular leak, 2 aortic dissection, 1 perforation of left ventricle			
Other	9	4 mitral valve disease, 3 CABG, 1 tricuspid valve, 1 ascending aortic aneurysm			
Total	31				

Abbreviations: CABG, coronary artery bypass grafting; CI, confidence interval; EuroSCORE: European System for Cardiac Operative Risk Evaluation; PCI, percutaneous coronary intervention; SAVR, surgical aortic valve replacement; TAVI, transcatheter aortic valve implantation.

**Figure 1.**
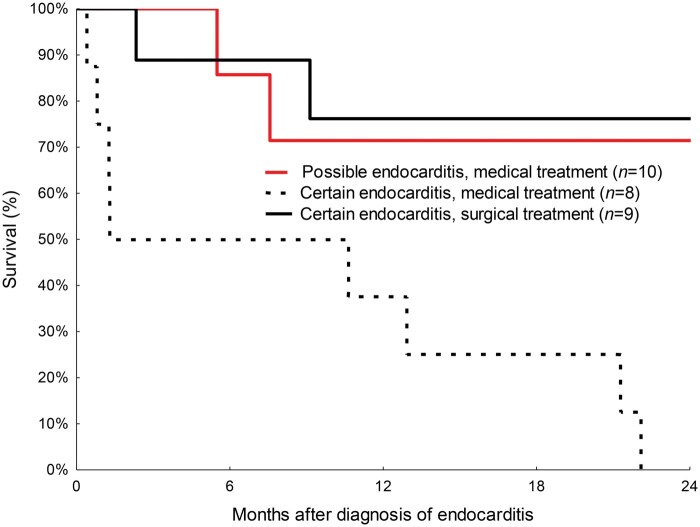
Survival after admission for endocarditis in patients with a previous transcatheter aortic valve implantation.

More than one-third of the infections occurred within the first year after TAVI. Of the 17 patients with definite endocarditis, 9 (53%) underwent aortic valve replacement. The EuroSCORE was high and there was 1 early death (11%) from bowel ischaemia and liver failure. The 2-year survival after an operation for definite endocarditis was 76% (**Figure 1**). Macroscopic findings of infection were observed in all the patients during the operations. Typically, the vegetation was located on the ventricular side of the aortic cusps. The valve device was explanted, the stenotic native calcified aortic valve was resected, and a prosthetic valve was inserted. The infection involved the prosthetic aortic valve and annulus in 2 patients with *Enterocccous faecalis* endocarditis who developed an aortic root abscess and underwent composite aortic root replacement: 1 with a mechanical valve and 1 with a porcine xenograft and a coronary artery implant. In 6 of the 9 explanted valves, 16S ribosomal DNA gene polymerase chain reaction analysis identified bacterial nucleic acids that aligned with preoperative blood cultures, with 3 each of *E faecalis* and *Streptococcus mitis*. Enterococci were cultured from the blood of 7 of the 27 patients (26%) with certain or possible endocarditis.

All 8 patients with definite endocarditis, who were not candidates for open-heart surgery because of severe comorbidities and frailty, died within 2 years. The cause of death was ongoing infection in 5 patients, malignancy in 2 and heart failure in 1 patient. Mild-to-moderate paravalvular leaks were observed in 7 of the 10 patients with possible endocarditis. The 2-year survival rate in this group was 72%.

### Non-infectious indications for heart surgery

Two years after open-heart surgery for causes other than infection following TAVI, the survival rate was 87% in 22 patients. These operations were performed at a median of 12 months (range, 1 day to 5 years) after TAVI. No deaths occurred within 2 years after the operations in the 3 patients who underwent surgery for structural valve deterioration. Patients with non-structural valve indications for surgery underwent either acute or urgent procedures. One patient who underwent aortic dissection during TAVI died of a myocardial infarction 6 days after an acute operation. The second patient was admitted for aortic dissection 6 days after TAVI and survived replacement of the ascending aorta with a graft. One patient died of heart failure 2 weeks after mitral valve replacement. The other 8 patients who underwent valve, coronary artery, or ascending aortic aneurysm surgery survived for 2 years.

## DISCUSSION

This study includes 31 patients who required open-heart surgery after TAVI. Surgical risk was associated with comorbidities, but not with previous TAVI per se. No major technical difficulties were encountered during explantation of the TAVI prosthesis.

Endocarditis was the indication for heart surgery in 9 of 31 (29%) patients. The diagnosis of endocarditis after TAVI is challenging. Transcatheter aortic valve implant endocarditis was classified as either definite or possible. In more than one-third of cases, admission due to endocarditis occurred within the first year after TAVI. The most common cause of infection in our series was *S mitis*, followed by *E faecalis* and *Staphylococcus aureus*, which is consistent with the findings of other reports.^8^^,^^9^ Gram-positive cocci were generally sensitive to our antibiotic prophylaxis. In contrast, Gram-negative enterococci tended to be resistant. Unfortunately, enterococci are common skin bacteria in adults. Prophylaxis with amoxicillin/clavulanic acid has been suggested.^8^ The reported incidence of endocarditis after TAVI is 0.2%-4.4%, and the mortality rate is high.^4^^,^^9^^,^^10^ Surgery for TAVI endocarditis is rarely performed.^4^^,^^9^ A nationwide Swedish study of all TAVI cases from 2008 to 2018 reported that the incidence of endocarditis was 1.4% during the first year. Of these, only 2 of 103 (1.9%) patients with endocarditis after TAVI underwent aortic replacement.^6^ Mangner et al.^5^ performed a registry analysis and found that the mortality rate was approximately 50% 1 year after admission, after both open-valve replacement and medical treatment in patients with endocarditis after TAVI.

### Limitations

Our series included too few patients to analyse risk factors for TAVI endocarditis. We did not follow all patients who underwent TAVI between 2016 and 2024. Elderly patients have an increased risk of infectious complications owing to hospitalization and comorbidities. Therefore, older patients with previous TAVI may have died of causes other than an infected valve, such as septicaemia.

## CONCLUSION

Patients may require open-heart surgery for various reasons after TAVI. Post-TAVI endocarditis is associated with high mortality. Surgical aortic valve replacement can be curative in selected patients with endocarditis after TAVI and also lifesaving after complications, such as aortic dissection and malpositioning of a prosthesis.

## Supplementary Material

ivaf173_Supplementary_Data

## Data Availability

Anonymized data underlying this article will be available on request to the corresponding author.
