# Supplementary material for: Endocarditis and other indications for open-heart surgery after a transcatheter aortic valve implant
Source: Interdiscip Cardiovasc Thorac Surg. 2025 Jul 26;40(8):ivaf173. doi: 10.1093/icvts/ivaf173 (PMC12349380; doi:10.1093/icvts/ivaf173)
Supplement: ivaf173_Supplementary_Data [file ivaf173_supplementary_data.zip › Supplementary Tables S1,S2.docx]

**Supplementay Table S1**: Type prosthesis in patients with endocarditis

|  | **Gender** | **Age (yrs)** | **TAVI year** | **Type of prosthesis** | **EuroSCORE** | **Heart surgery year** |
| --- | --- | --- | --- | --- | --- | --- |
| *Definite endocarditis, surgery performed* | | | | |  |  |
|  | Male | 71 | 2016 | ACURATE | 73.7 | 2023 |
|  | Male | 61 | 2018 | SAPIEN | 3.3 | 2024 |
|  | Male | 62 | 2019 | SAPIEN | 19.7 | 2019 |
|  | Male | 81 | 2019 | SAPIEN | 3.4 | 2020 |
|  | Male | 81 | 2019 | SAPIEN | 3.1 | 2021 |
|  | Male | 79 | 2020 | SAPIEN | 10.4 | 2021 |
|  | Female | 74 | 2020 | SAPIEN | 32.5 | 2022 |
|  | Male | 62 | 2021 | SAPIEN | 1.1 | 2023 |
|  | Male | 75 | 2023 | SAPIEN | 17.0 | 2022 |
| *Definite endocarditis, medical treatment* | | | | |  |  |
|  | Female | 77 | 2018 | Evolut | 4.3 |  |
|  | Female | 47 | 2018 | ACURATE | 9.3 |  |
|  | Male | 80 | 2020 | Evolut | 12.1 |  |
|  | Female | 76 | 2020 | ACURATE | 5.8 |  |
|  | Male | 80 | 2020 | ACURATE | 15.4 |  |
|  | Male | 69 | 2020 | LOTUS | 3.2 |  |
|  | Male | 80 | 2022 | ACURATE | 7.0 |  |
|  | Female | 88 | 2024 | ACURATE | 11.0 |  |
| *Possible endocarditis, medical treatment* | | | | |  |  |
|  | Male | 84 | 2017 | SYMETIS | 7.2 |  |
|  | Male | 76 | 2019 | Evolut | 11.0 |  |
|  | Male | 66 | 2020 | ACURATE | 9.2 |  |
|  | Male | 81 | 2021 | SAPIEN | 7.5 |  |
|  | Female | 85 | 2022 | ACURATE | 4.9 |  |
|  | Male | 86 | 2023 | ACURATE | 5.1 |  |
|  | Male | 76 | 2023 | ACURATE | 3.9 |  |
|  | Female | 88 | 2023 | ACURATE | 8.8 |  |
|  | Male | 80 | 2023 | Medtronic pro | 4.3 |  |
|  | Male | 81 | 2024 | Evolut | 4.3 |  |

**Supplementary Table S2**: Noninfectious indication for open heart surgery after previous transcatheter aortic valve implantation (*n*=22)

| **Causes** | **No.** | **Age** | **Gender** | **TAVI (yr)** | **Prosthesis** | **Indication for surgery** | **Euro SCORE** | **Heart surgery (yr)** |
| --- | --- | --- | --- | --- | --- | --- | --- | --- |
| Structural valve deterioration | 3 | 82 | Female | 2016 | Evolut | Cusp rupture | 11.4 | 2021 |
|  |  | 76 | Female | 2018 | Evolut | Prosthesis stenosis | 23.7 | 2022 |
|  |  | 62 | Female | 2022 | Sapien | Prosthesis stenosis | 2.9 | 2024 |
| Non-structural valve deterioration | 10 | 81 | Male | 2021 | ACURATE | Aortic dissection | 84.6 | 2021 |
|  |  | 79 | Male | 2024 | Evolut | Aortic dissection | 66.8 | 2024 |
|  |  | 79 | Male | 2022 | ACURATE | Supraanular position | 9.96 | 2022 |
|  |  | 77 | Female | 2024 | ACURATE | Subanular position | 6.19 | 2024 |
|  |  | 73 | Male | 2023 | ACURATE | Subanular position | 10.4 | 2023 |
|  |  | 72 | Male | 2023 | ACURATE | Subanular position | 69.0 | 2023 |
|  |  | 58 | Male | 2023 | Sapien | Subanular position | 16.1 | 2023 |
|  |  | 76 | Male | 2020 | Sapien 3 | Paravalvular leak | 31.1 | 2020 |
|  |  | 73 | Male | 2019 | ACURATE | Paravalvular leak | 35.1 | 2021 |
|  |  | 78 | Male | 2024 | Sapien | Perforation of left ventricle | 21.58 | 2024 |
| Other | 9 | 75 | Female | 2018 | SYMETIS | Mitral valve insufficiency | 5.5 | 2023 |
|  |  | 84 | Female | 2022 | ACURATE | Mitral valve insufficiency | 20.6 | 2023 |
|  |  | 76 | Female | 2021 | ACURATE | Mitral valve stenosis | 12.56 | 2021 |
|  |  | 75 | Female | 2022 | ACURATE | Mitral valve stenosis | 14.1 | 2024 |
|  |  | 78 | Male | 2022 | Sapien | Coronary artery bypass | 8.13 | 2024 |
|  |  | 76 | Male | 2018 | ACURATE | Coronary artery bypass | 4.27 | 2022 |
|  |  | 76 | Male | 20221 | Sapien | Coronary artery bypass | 10.8 | 2022 |
|  |  | 76 | Male | 2017 | Sapien | Tricuspid insufficiency | 17.6 | 2022 |
|  |  | 78 | Male | 2019 | Evolut | Ascending aortic aneurysm | 48.3 | 2023 |
