# Supplementary figures and images for: Endocarditis and other indications for open-heart surgery after a transcatheter aortic valve implant
Source: Interdiscip Cardiovasc Thorac Surg. 2025 Jul 26;40(8):ivaf173. doi: 10.1093/icvts/ivaf173 (PMC12349380; doi:10.1093/icvts/ivaf173)

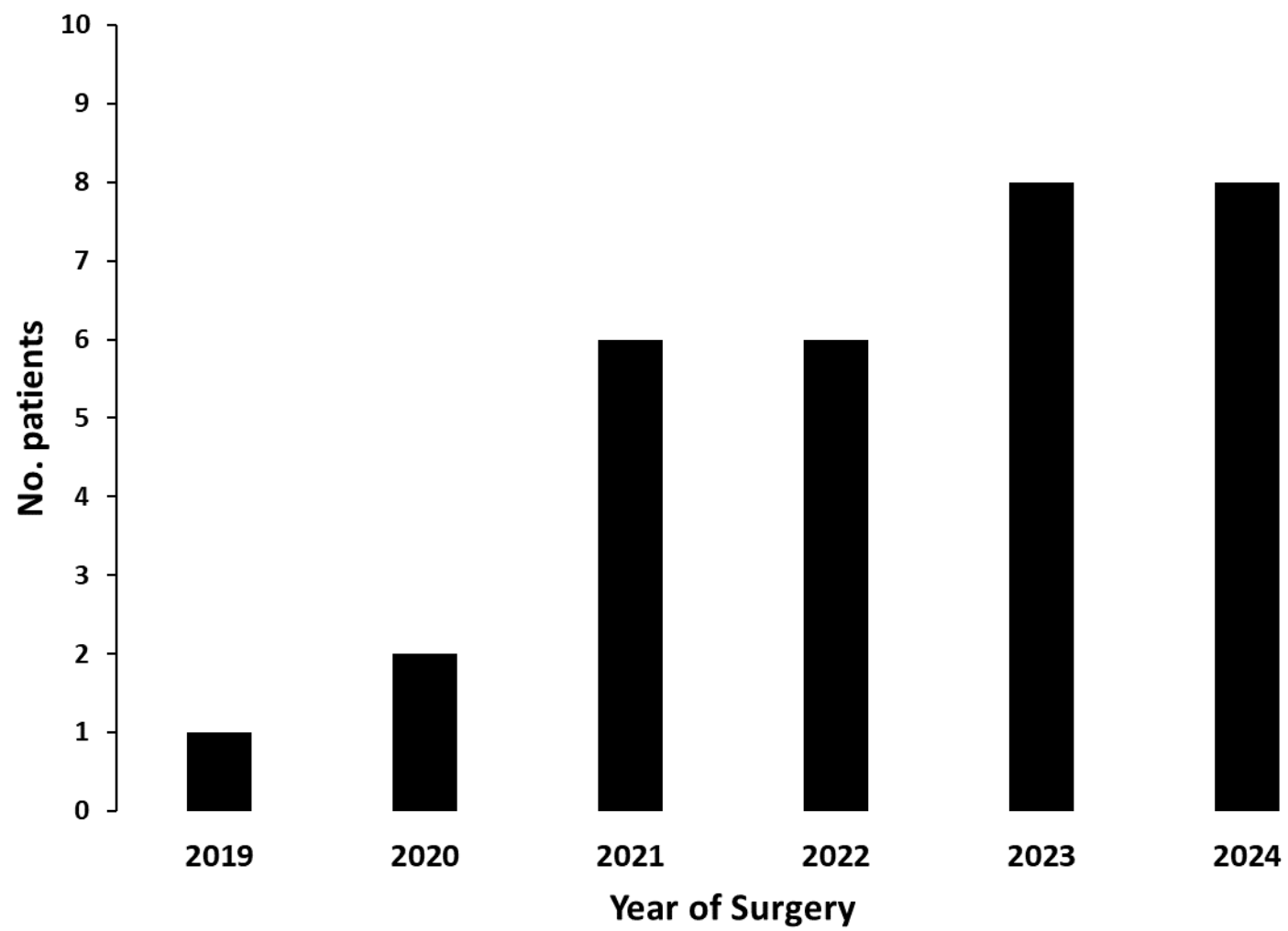

Supplement: ivaf173_Supplementary_Data [file ivaf173_supplementary_data.zip › Supplementary Figure S1.pdf]
